# Direct Laser Writing of Magneto-Photonic Sub-Microstructures for Prospective Applications in Biomedical Engineering

**DOI:** 10.3390/nano7050105

**Published:** 2017-05-09

**Authors:** Thi Huong Au, Duc Thien Trinh, Quang Cong Tong, Danh Bich Do, Dang Phu Nguyen, Manh-Huong Phan, Ngoc Diep Lai

**Affiliations:** 1 Laboratoire de Photonique Quantique et Moléculaire, UMR 8537, École Normale Supérieure de Cachan, Centrale Supélec, CNRS, Université Paris-Saclay, 61 avenue de Président Wilson, 94235 Cachan, France; huongauthibn@gmail.com (T.H.A.); congtq2004@yahoo.com (Q.C.T.); 2 Faculty of Physics, Hanoi National University of Education, 136 Xuan Thuy, Cau Giay, 100000 Hanoi, Vietnam; thientd@hnue.edu.vn (D.T.T.); dodanhbich@gmail.com (D.B.D.); phund@hnue.edu.vn (D.P.N.); 3 Institute of Materials Science, Vietnam Academy of Science and Technology, 18 Hoang Quoc Viet, Cau Giay, 100000 Hanoi, Vietnam; 4 Department of Physics, University of South Florida, Tampa, FL 33620, USA; phanm@usf.edu

**Keywords:** magnetic nanocomposite, magneto-photonic microstructures, one-photon absorption direct laser writing

## Abstract

We report on the fabrication of desired magneto-photonic devices by a low one-photon absorption (LOPA) direct laser writing (DLW) technique on a photocurable nanocomposite consisting of magnetite (${\mathrm{Fe}}_{3}$$O_{4}$) nanoparticles and a commercial SU-8 photoresist. The magnetic nanocomposite was synthesized by mixing ${\mathrm{Fe}}_{3}$$O_{4}$ nanoparticles with different kinds of SU-8 photoresists. We demonstrated that the degree of dispersion of ${\mathrm{Fe}}_{3}$$O_{4}$ nanoparticles in the nanocomposite depended on the concentration of ${\mathrm{Fe}}_{3}$$O_{4}$ nanoparticles, the viscosity of SU-8 resist, and the mixing time. By tuning these parameters, the most homogeneous magnetic nanocomposite was obtained with a concentration of about 2 wt % of ${\mathrm{Fe}}_{3}$$O_{4}$ nanoparticles in SU-8 2005 photoresist for the mixing time of 20 days. The LOPA-based DLW technique was employed to fabricate on demand various magneto-photonic submicrometer structures, which are similar to those obtained without ${\mathrm{Fe}}_{3}$$O_{4}$ nanoparticles. The magneto-photonic 2D and 3D structures with sizes as small as 150 nm were created. We demonstrated the strong magnetic field responses of the magneto-photonic nanostructures and their use as micro-actuators when immersed in a liquid solution.

## 1. Introduction

Nanomaterials with excellent optical, electronic, and magnetic properties are highly desirable for fabrication of functional photonic structures and devices. Various kinds of nanofillers, such as carbon nanostructures [1], metallic [2], and semiconducting nanoparticles [3], are selected to incorporate into polymer matrices for creating functional nanocomposites with hybrid properties. In particular, magneto-polymer nanocomposites have attracted a great attention due to their multifunctionality for a variety of applications, ease of processability and potential for large-scale manufacturing [4,5,6,7,8].

Structures made by magnetic nanocomposites are useful for a wide range of applications including data storage [9,10], sensors [11], actuators [12], biomedicine [13], etc. A number of synthesis and fabrication methods have been proposed to obtain desired magnetic structures, for instance, self-assembly [14,15], bio-templating [16], inkjet-printing [17], and stop-flow lithography [18]. However, these methods are complicated, expensive, and usually refer to large-scale structures. It causes challenges when further miniaturization devices are desired, for example, minimal invasive surgery [19], targeted drug delivery [20], and remote manipulation on a single cell [21].

Direct laser writing (DLW) is a well-developed optical lithography technique allowing fabrication of multi-dimensional polymeric nanostructures [22,23]. In this technique, a laser beam is tightly focused into a photosensitive polymer material, called photoresist, to locally induce a polymerization effect. By moving the focusing spot, where the light intensity is high enough to initiate or to induce polymerization, following a programmed trajectory and after the development process that washes out the unexposed area, the desired polymeric structures are obtained. This DLW employing two-photon absorption (TPA) mechanism has recently been proposed to realize some magnetic devices on demand [24,25]. It is worth mentioning that while the TPA-based DLW technique is very powerful for fabrication of desired structures, it requires expensive laser sources and delicate specialized optics. To overcome this drawback, a simple and low-cost method called low one-photon absorption (LOPA) DLW [26,27], which has advantages of both OPA and TPA DLW, has recently been demonstrated. This technique has enabled the fabrication of one-, two-, and three-dimensional (1D, 2D, and 3D) structures with a feature size as small as 150 nm, by using a simple continuous-wave laser at 532 nm with only a few milliwatts.

In this work, we demonstrated the use of LOPA-based DLW as a simple and robust technique for the fabrication of desired 1D, 2D, and 3D magneto-photonic structures with sub-wavelength size. We first elaborated a photocurable nanocomposite consisting of magnetic nanoparticles (MNPs) and a commercial SU-8 photoresist. The concentration of ${\mathrm{Fe}}_{3}$$O_{4}$ MNPs, the viscosities of SU-8 photoresist and the preparation time were adjusted to obtain a uniformly dispersed magnetic nanocomposite. We then successfully fabricated a variety of desired magneto-photonic structures and demonstrated the manipulation of these free-floating nanostructures by applying an external magnetic field. This shows a strong and controllable magnetic field response of the fabricated magneto-photonic structures, paving the way for different applications, such as biosensors [28], actuators, and magnetic labeling [12].

## 2. Synthesis of Magneto-Polymer Composites

**Figure A1:**
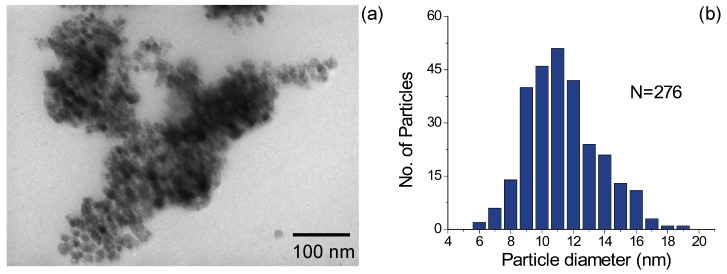
(**a**) TEM image of magnetite nanoparticles (Fe${}_{3}$O${}_{4}$) in ethanol; (**b**) statistical results of Fe${}_{3}$O${}_{4}$ size distribution.

**Figure A2:**
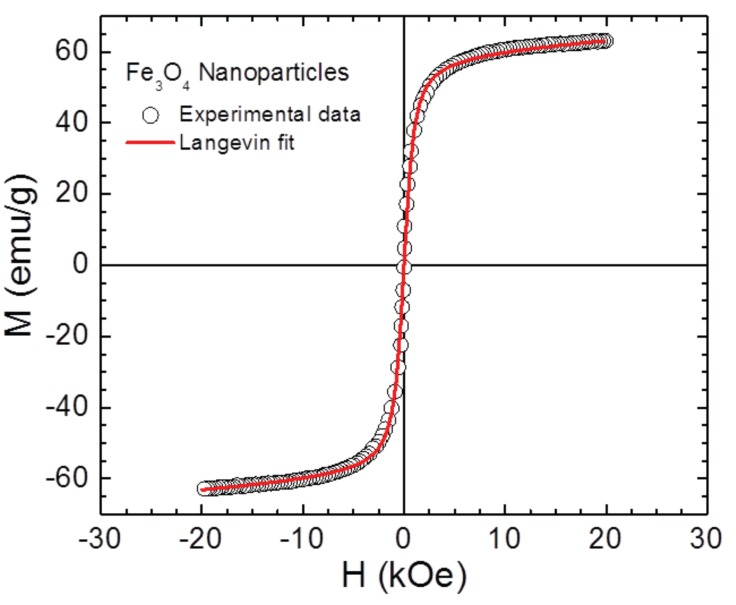
Magnetic hysteresis loop M(H) of Fe${}_{3}$O${}_{4}$ nanoparticles taken at 300 K. The solid line shows the best fit of the M(H) data using the Langevin function.

**Figure A3:**
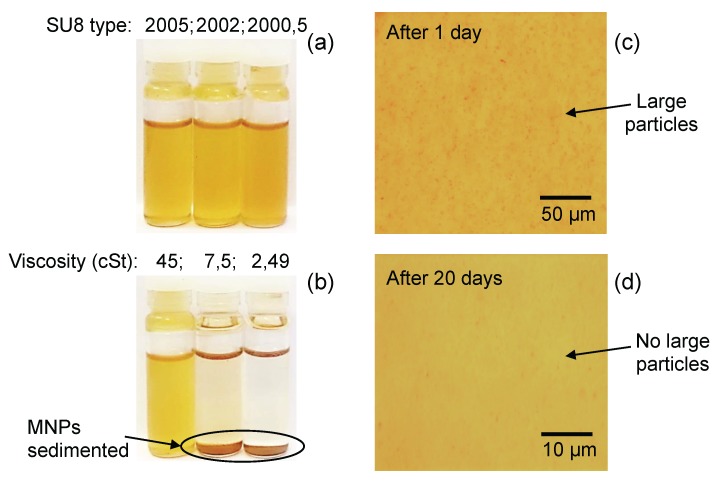
The dispersion of Fe${}_{3}$O${}_{4}$ nanoparticles in SU-8 2000.5, SU-8 2002, and SU-8 2005. (**a**) images of three bottles of nanocomposites solutions, taken just after mixing; (**b**) after a few hours, the sedimentation occurred in the nanocomposite solution having a low viscosity of 2.49 cSt and 7.5 cSt; zoom in image of the bottle containing the Fe${}_{3}$O${}_{4}$/SU-8 2005 nanocomposite, after (**c**) one day and (**d**) 20 days of the preparation.

The photopatternable magneto-polymer nanocomposite consists of ${\mathrm{Fe}}_{3}$$O_{4}$ nanoparticles with an average diameter of 11 nm (determined from transmission electron microscopy (TEM), see Figure A1 in Appendix A), dispersed in SU-8 negative tone photoresist. Details of the synthesis of ${\mathrm{Fe}}_{3}$$O_{4}$ MNPs are reported in the Experimental section. Magnetic hysteresis loop was also performed on the dried ${\mathrm{Fe}}_{3}$$O_{4}$ nanoparticles at 300 K in order to confirm the room temperature superparamagnetic characteristic of these MNPs. The data (Figure A2) and relevant discussions are presented in Appendix A. The original MNPs were suspended in ethanol. Due to their nature of being magnetic forces (mainly inter-particle interactions), they tend to form large agglomerations, causing difficulties with achieving a homogeneous distribution of MNPs in polymer matrices. For this reason, MNPs in ethanol were sonicated for 30 min before incorporating them into a polymer matrix. It has been shown that if the particle–polymer interactions can be increased relative to the polymer–polymer and particle–particle interactions, the particles will uniformly disperse in the polymer matrice [4,29,30,31]. Therefore, in the present study, various types of epoxy-based negative photoresist, SU-8 2000.5, SU-8 2002, SU-8 2005, SU-8 2010, and SU-8 2025 (MicroChem Corp., Westborough, MA 01581 USA) with different viscosities of 2.49 cSt, 7.5 cSt, 45 cSt, 380 cSt, and 4500 cSt, respectively, were used as candidates for hosting MNPs. Different concentrations of ${\mathrm{Fe}}_{3}$$O_{4}$ MNPs in SU-8 ranging from 0 to 10 wt % were also investigated. First, we found that the SU-8 2005 possessing a moderate viscosity of 45 cSt was the most appropriate host for obtaining well-dispersed and uniformly-suspended MNPs, while the MNPs quickly sedimented in photoresists having low viscosity, such as SU-8 2000.5 and SU-8 2002, and it was very difficult to mix MNPs with photoresists having very high viscosity, such as SU-8 2010 and SU-8 2025. Secondly, the most homogeneous magnetic nanocomposite was obtained with a modest concentration of ${\mathrm{Fe}}_{3}$$O_{4}$ MNPs of about 2 wt % and lower. For higher concentrations, the nanocomposite solutions showed some large magnetic agglomerations, and they tended to get together at the bottom of the bottle containing the nanocomposite solution. In order to demonstrate the homogeneous dispersion of MNPs in SU-8 photoresist, the transmission electron microscope (TEM) could be an excellent method [32,33]; however, this TEM equipment is not available in our laboratory at this moment. Nevertheless, the homogeneous dispersion of MNPs was indirectly confirmed by the fabricated structures, as shown in the next. Indeed, the fabrication of micro and larger structures has shown that the structure surfaces are very rough due to the non-uniformity of the nanocomposite. We thus concluded that the best magnetic nanocomposite was obtained in SU-8 2005 with an MNP concentration of about 2 wt %, where it is low enough to achieve a homogeneous nanocomposite and high enough to give strong magnetic field responses. It was also found experimentally that the dispersion was remarkably improved by the preparation time. The ${\mathrm{Fe}}_{3}$$O_{4}$/SU-8 2005 nanocomposite was stored in a nonmagnetic environment before the fabrication and was checked daily by an optical microscope. It was shown that big clusters observed in the nanocomposite right after mixing were no longer seen in the same nanocomposite after several days (see the optical images of these samples in Figure A3 in Appendix A). Furthermore, the microstructures fabricated by the nanocomposite right after mixing exhibited a rough surface, while those fabricated by the nanocomposite prepared for 20 days prior to the fabrication possessed a very smooth surface. It proves that the viscosity of the host medium and the preparation time had strong impacts on the inter-diffusion of the MNPs in suspension, which resulted in the homogeneous distribution of the nanoparticles without using any surfactant agent. This has enabled us to fabricate novel magneto-optical structures at nanoscale, as shown below.

## 3. Realization of Magneto-Photonic Structures by LOPA-Based DLW Technique

### 3.1. Working Principle of LOPA-Based DLW

**Figure 1:**
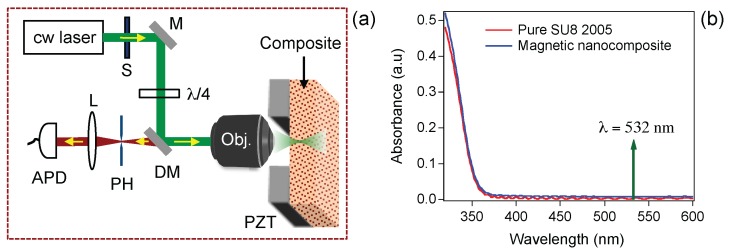
(**a**) experimental setup of a LOPA-based DLW technique for realization of submicrometer magneto-photonic structures; (**b**) a comparison of absorption spectra of pure SU-8 2005 and Fe${}_{3}$O${}_{4}$/SU-8 2005 nanocomposite.

DLW is an important and relevant technique to fabricate 2D and 3D structures and devices with arbitrary shapes at submicrometer resolution. In LOPA-based DLW technique [26,34], a green (532 nm) laser beam is focused into a negative SU-8 photoresist resulting in a polymerized and insoluble voxel. By controlling the focusing position/area, the desired structures could be created. As compared to TPA-based DLW technique [22,23], the LOPA is simpler, more compact, and less expensive [26,27]. The LOPA-based DLW experimental setup is shown in Figure 1a. It is interesting to mention (see Figure 1b) that the absorption of the magnetic nanocomposite is very similar to that of pure SU-8 photoresist, as a result of low concentration of ${\mathrm{Fe}}_{3}$$O_{4}$ MNPs (2 wt %). This fact is very important for fabrication of submicrometer structures in 2D and 3D using this nanocomposite. The absorption spectrum indicates the ultralow absorption at excitation wavelength of 532 nm. Therefore, the DLW used for the ${\mathrm{Fe}}_{3}$$O_{4}$/SU-8 2005 nanocomposite operates in the LOPA regime. Based on this advantage, we have then demonstrated the realization of 2D and 3D magneto-polymer submicrostructures by LOPA-based DLW.

### 3.2. Controlling Structure Size with Exposure Power and Scanning Velocity

**Figure 2:**
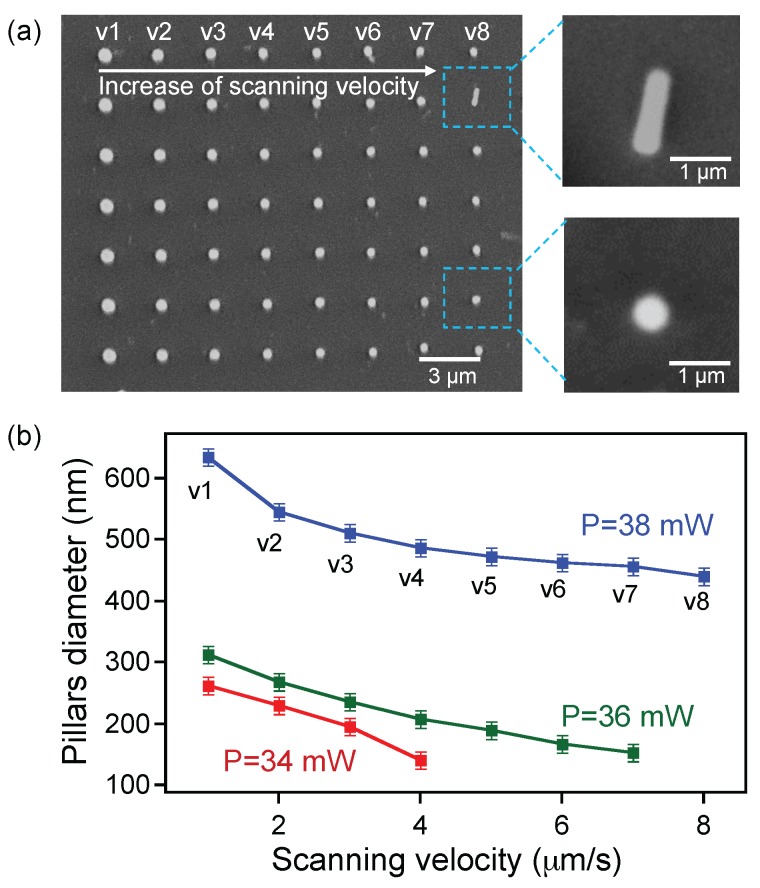
Dependence of the size of magneto-photonic pillars on exposure doses (laser power and scanning velocity). (**a**) the SEM image of a periodic pillars array with different diameters realized by different scanning velocities (the laser power was fixed at 38 mW). Images on the right show side and top views of a single pillar; (**b**) plot of pillar diameters as a function of scanning speed for three different exposure powers.

In the LOPA operation regime, the excitation power and exposure time are important parameters, which directly determine the characterization of fabricated structures. To study the behavior of the photopatternable magnetic nanocomposite, we first investigated the evolution of structure size as a function of laser power and writing speed. Series of submicropillars were fabricated on a film thickness of 2 μm and analyzed to obtain a size distribution on each fabrication parameter. We note that, in the DLW, a single exposure results in a single polymerized voxel, and a pillar is obtained by scanning the focusing spot along the film thickness. Figure 2a shows an SEM image of a 2D periodic structure constituted of different pillars, which were created when using different writing speeds, ranging from 1 μm/s to 8 μm/s, and a laser power of 38 mW. The pillar diameter changes as a function of the scanning speed, and very smaller pillars could be obtained with this magnetic nanocomposite, as shown in the left images of Figure 2a. With a laser power of 38 mW, the pillar size varies between 400 nm and 650 nm, as shown in Figure 2b. By decreasing the laser power to 36 mW or 34 mW, the pillar sizes decreased further to a range of 150 nm–300 nm. Due to the ultralow absorption effect, it was not possible to obtain polymerized structures with further decrease of the laser power and with a reasonable writing speed. This result shows that the fabricated structures on magnetic nanocomposite are quite similar to those obtained by pure SU-8 photoresist [26]. By performing many different tests, we have found that the laser power of 36 mW and the scanning velocity of 4 μm/s allow for obtaining small and stable submicropillars. Furthermore, the smallest feature size of 150 nm is even smaller than the minimum focusing spot (a diffraction limit) of the used objective lens. Obviously, the ${\mathrm{Fe}}_{3}$$O_{4}$/SU-8 2005 nanocomposite with a low particle concentration of 2 wt % is an excellent hybrid material to create magnetic nanostructures by the LOPA-based DLW technique. The strong magnetic interactive responses of these nanopillars will be demonstrated in Section 4.

### 3.3. Influence of Structure Period on Structure Size

**Figure 3:**
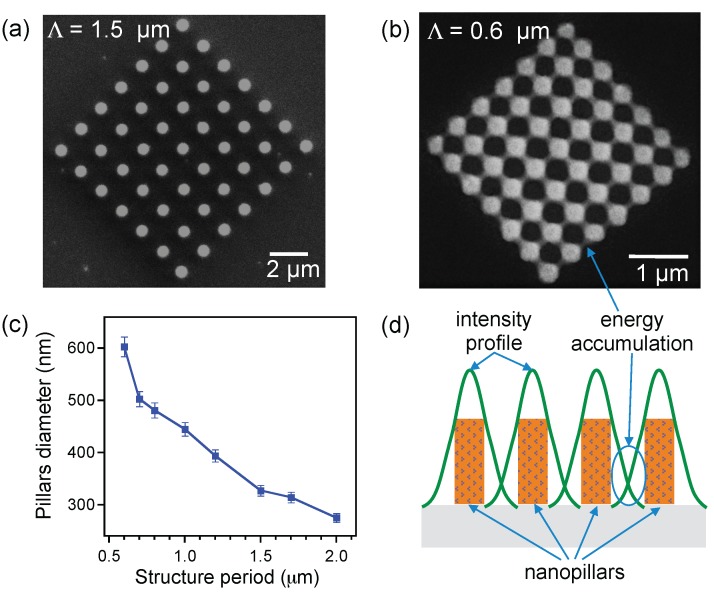
Influence of the dose accumulation effect on pillars sizes. SEM images of periodic pillars arrays realized with a period of 1.5 μm (**a**) and with a period of 0.6 μm (**b**); (**c**) pillar diameters as a function of the distance between pillars (the period of the array). The laser power and the writing speed were fixed at 36 mW and 4 μm/s, respectively, for all pillars; (**d**) schematic illustration of the dose accumulation effect due to the short distance between two pillars, which creates a nano-connection between pillars.

For most applications of photonic structures, the small size is not only a key parameter but also the minimum distance between two features. We have therefore examined the quality of structures as a function of the structure lattice. Different magneto-photonic structures with a periodicity varying from 2 μm to 0.4 μm were fabricated and depicted, for example, in Figure 3a,b. The size of pillars increased when the distance between pillars decreased as shown in Figure 3c. We found that the good structures were obtained with a periodicity larger than 0.5 μm, since the microscope objective has a numerical aperture of 0.9. Actually, the fabrication relies on an OPA mechanism, and there exists a dose accumulation effect with small periodicity [26,34]. The degree of polymerization within the laser spot as well as in the out-of-focus region are completely defined by the number of photons absorbed linearly during exposure, which leads to unwanted structures when pillars are set very close to each other. The mechanism of this accumulation effect is illustrated in Figure 3d. This result is again similar to that observed with pure SU-8 photoresist [34]. In addition, the optically induced thermal effect became more pronounced with the presence of MNPs, which exhibit a thermosensitive property. This thermal effect accelerated the polymerization effect and locally heated up the exposed area, and the post-exposure bake (PEB) process was therefore neglected [35]. Thanks to the local PEB, the minimum distance (0.5 μm) between pillars is even better realized than that with pure SU-8 photoresist, by using the same optical setup. The small size and short distance should be good enough for different applications based on magneto-photonic structures.

### 3.4. Realization of Desired 2D and 3D Magneto-Photonic Structures

**Figure 4:**
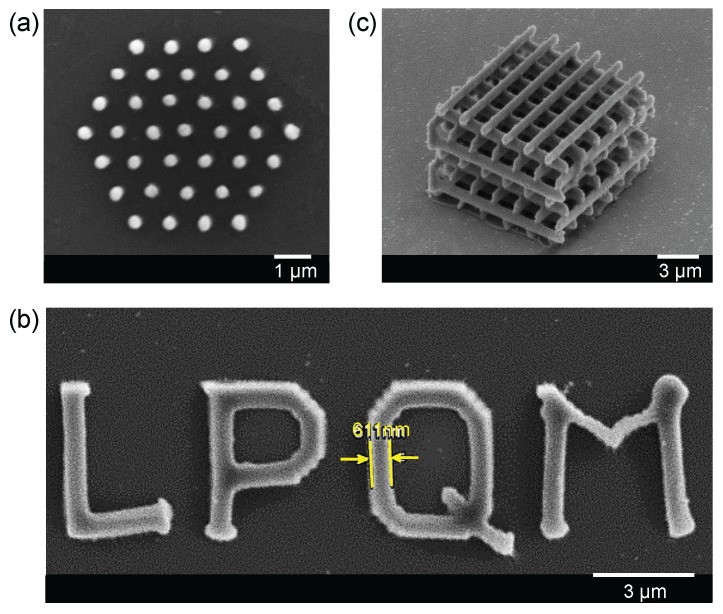
SEM images of various magneto-photonic submicrometer structures fabricated by the LOPA-based DLW technique. (**a**) a 2D hexagonal structure with a period of 1 μm; (**b**) an arbitrary “LPQM” letter; (**c**) a 3D woodpile structure.

The great advantage of the DLW method is that it allows one to realize any structure on demand. Based on the above investigations of structure size and periodicity, we have demonstrated the use of a LOPA-based DLW technique to pattern arbitrary magnetic structures by using the magneto-polymer nanocomposite. The fabrication parameters were chosen appropriately for each design. Figure 4a shows an SEM image of a 2D hexagonal magneto-photonic structure, which has a period of 1 μm. This structure was fabricated by a laser power of 36 mW and a writing speed of 4 μm/s. Figure 4b shows an SEM image of an arbitrarily shaped 2D structure, the letter ”LPQM”. For this fabrication, we have used a point matrix technique to shape the letter. The distance between pillars was set at 150 nm, which resulted in a continuous line due to the accumulation of exposure energy at the vicinity of each point. For creating this continuous structure, a laser power of about 15 mW was needed, which is lower than that used for fabrication of independent pillars. This is due to the thermal effect of MNPs, as explained previously. Similarly, various 3D structures have also been realized, with a modest laser power of about 13 mW. Figure 4c shows an SEM image of a woodpile structure, which is made by 10 alternative layers separated to each other by 1 μm. The distance between two lines in *x*- and *y*-directions is 2 μm. All of these experimental results confirm that any magneto-photonic submicrometer structure or device can be realized by using the LOPA-based DLW on this magneto-polymer nanocomposite. It opens a possibility to go further on development of magnetic nano-devices and micro-robotic tools for a wide range of applications.

## 4. Reversible Magnetic Field-Driven Motion of Magneto-Photonic Devices

**Figure 5:**
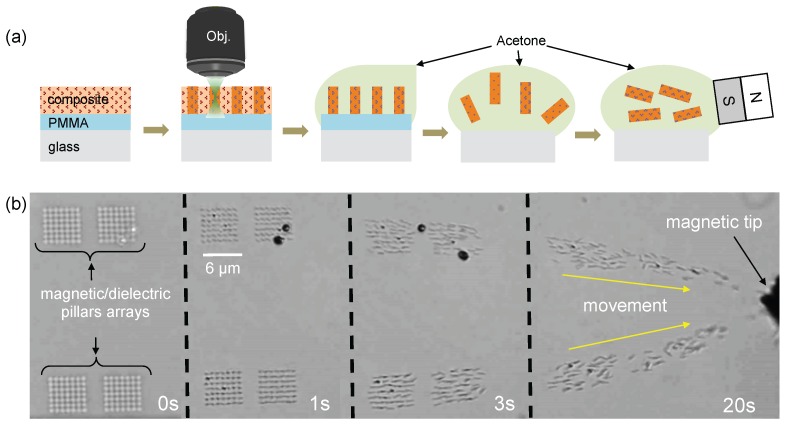
(**a**) illustration of the fabrication process and (**b**) series of screenshots illustrating the movement of magnetic submicropillars (the diameter is about 300 nm) towards the magnetic tip (see videos S.2 in the Supplementary Materials).

Exploiting reversible magnetic field-driven motion of magneto-photonic structures for remote actuation in biomedical applications has recently attracted a growing attention [36]. With the aid of an external magnetic field, it is possible to control the displacement of magnetic structures in three dimensions as desired. The challenge is how to create small magnetic structures that are adaptable to small targets. As demonstrated in the previous section, the small magneto-photonic structures have been created. Since small pillars contain ${\mathrm{Fe}}_{3}$$O_{4}$ MNPs, an important question arises as to how these tiny structures respond to an applied external magnetic field. We have used LOPA-based DLW to realize free-floating magnetic nanostructures as a remote control nanodevice. Submicropillar arrays were fabricated as an example for demonstration. In order to release the structures from the substrate, an extra sacrificial layer of PMMA (Microchem Corp. Westborough, MA 01581 USA) was added on glass substrate before coating the nanocomposite layer, as illustrated Figure 5a. After the exposure step, the PMMA layer can be dissolved by acetone to release structures into solution. A magnetic field (only 8 mT) generated by a permanent magnet was then applied to examine the magnetic field response of the magnetic submicropillars (diameter, 300 nm). Figure 5b shows a series of screenshots to illustrate the whole process from structural development to their movement toward higher gradient of the external magnetic field (see videos in the Supplementary Materials). Obviously, all of the fabricated submicropillars quickly moved toward the magnetic tip, confirming the presence of ${\mathrm{Fe}}_{3}$$O_{4}$ MNPs inside nanopillars and their strong response to the applied magnetic field. The translational motion was the main activity as recorded when the nanopillars were subjected to a magnetic field gradient. The rotational movement was hardly observed due to the small size and symmetric shape of pillars. It is also possible to perform other activities using submicro-structures with different size and shape of structures. These observations underline the importance of free-floating structures as a robotic technology for magnetic devices such as sensors [11], actuators [12], magnetic labeling, and drug targeting [20].

Finally, we expect that it is possible to apply an external magnetic field with tunable magnitude and orientation at the position of the focusing spot during the fabrication process by using a using Helmholtz coils system, in order to realize a magneto-photonic structure with controllable magnetic response of individual feature. Examples of these structures include magnetic vortex, 3D magnetic spiral structures, etc. These magneto-photonic structures will be very interesting for applications in tunable photonic devices or data storage, etc.

## 5. Experimental Section

### 5.1. Magnetite Nanoparticle Preparation

The ${\mathrm{Fe}}_{3}$$O_{4}$ (magnetite) nanoparticles were chemically synthesized as the following: a magnetic fluid was prepared by using the conventional precipitation of ${\mathrm{Fe}}^{3+}$ and ${\mathrm{Fe}}^{2+}$ ions by ${\mathrm{OH}}^{-}$ at room temperature. In a typical case, 4.17 g of ${\mathrm{FeCl}}_{3}$·6$H_{2}$O and 1.52 g of ${\mathrm{FeCl}}_{2}$·4$H_{2}$O were dissolved in 80 mL of distilled water with stirring. Then, a solution of 6 mL of 35% ${\mathrm{NH}}_{4}$OH was added at a rate of 1 drop per second during a constant stirring. Black precipitates of ${\mathrm{Fe}}_{3}$$O_{4}$ were formed and isolated from the solution by the magnetic decantation method. The water washing and decantation process were repeated several times to purify MNPs. The average size of MNPs is determined to be about 11 nm using electron transmission microscopy (see Figure A1 in Appendix A).

### 5.2. Elaboration of Magnetic Nanocomposites

In order to mix ${\mathrm{Fe}}_{3}$$O_{4}$ MNPs with SU-8 photoresist, the magnetic decantation process was repeated to exchange solution from water to ethanol. The ${\mathrm{Fe}}_{3}$$O_{4}$ MNPs in ethanol were treated with an ultrasonic wave in a conventional ultrasonic-bath within 30 min before introducing them into a polymer matrix. Different types of epoxy-based negative photoresist, SU-8 2000.5, SU-8 2002, SU-8 2005, SU-8 2010, and SU-8 2025 (MicroChem Corp.) with different viscosities of 2.49 cSt, 7.5 cSt, 45 cSt, 380 cSt, and 4500 cSt, respectively, were used as candidates for hosting MNPs. The dispersion of ${\mathrm{Fe}}_{3}$$O_{4}$ MNPs in SU-8 photoresist was also investigated with different concentrations of ${\mathrm{Fe}}_{3}$$O_{4}$ MNPs ranging from 0 to 10 wt %. The mixtures were stirred for 2 h (typical duration), followed by 30 min of sonication. Finally, the nanocomposite solutions of different viscosities were stored in a nonmagnetic environment and examined by optical microscopy as a function of time.

### 5.3. Nanocomposite Thin Film Preparation Process

Firstly, the glass substrates were pretreated with sonication in acetone within 15 min to eliminate all the surface contamination, soaked in isopropanol to remove exceeding acetone and then rinsed in distilled water. These substrates were finally dried by a Nitrogen gas, followed by 1 min on a hot plate at $180{\;}^{\circ }$C. The thin film of material was obtained by spin-coating the nanocomposite on cleaned substrates following the recommended parameters of pure SU-8 resist. For example, to obtain a thin film of 5 μm–thickness using ${\mathrm{Fe}}_{3}$$O_{4}$/SU-8 2005 nanocomposite, the spin-coating parameters were set as: speed = 3000 rpm, acceleration = 300 rpm/s, and duration = 60 s. To remove the residual solvent, the samples were then put on a hot plate for 1 min at $65{\;}^{\circ }$C and 3 min at $95{\;}^{\circ }$C. Note that, for fabrication of free-floating magneto-photonic structures, the glass substrates were covered by a uniform layer of PMMA (500 nm–thickness) by the same spin-coating method before the spin-coating nanocomposite layer. The samples must be protected from room light to avoid undesired solidification of the nanocomposite. Samples were then used to fabricate desired magneto-optical structures by LOPA-based DLW.

### 5.4. Fabrication and Development Procedure of Structures

The DLW experimental setup is illustrated in Figure 1a. A continuous-wave (cw) green laser beam (λ = 532 nm) was tightly focused into the sample by an air-immersion (NA = 0.9) objective lens. The sample was translated in 3D space following a controllable trajectory by a high-resolution piezo translation (PZT) stage. The structures are fabricated by different powers (from 10 μW to 40 mW) and different scanning speeds, from 1 μm/s to 8 μm/s. Thanks to the optically induced thermal effect of the nanocomposite in OPA regime [35], the samples were developed right after the exposure without the post-exposure baking process that is usually required when using a standard TPA-based DLW. For development of structures, the exposed samples were emerged in SU-8 developer, followed by isopropanol and distilled water for 2 min for each step to get rid of unexposed parts, leaving desired structures on glass or PMMA-covered glass substrates.

## 6. Conclusions

In this work, we have successfully demonstrated the fabrication of magneto-photonic sub-microstructures on demand utilizing the LOPA-based DLW technique. By incorporating magnetite nanoparticles into SU-8 polymer matrix with different viscosities, we have found that SU-8 2005 with a viscosity of 45 cSt was the most appropriate photoresist for hosting MNPs. Different concentrations of ${\mathrm{Fe}}_{3}$$O_{4}$ MNPs were analyzed, and the concentration of about 2 wt % was found to be the best to achieve a uniform distribution of nanoparticles in SU-8 without any sedimentation and surfactant agent. The sample preparation time also played an important role in promoting the particle dispersion, thus enabling the fabrication of high quality magnetic nanostructures. The LOPA-based DLW technique has been successfully employed to realize desired 2D and 3D magneto-photonic submicropatterns, which are similar to those realized by using pure SU-8 photoresist. The magneto-photonic structure size and periodicity were analyzed as a function of exposure power and scanning velocity. The smallest structure size of about 150 nm and the minimum structural distance of 500 nm are achieved. Furthermore, due to the thermal effect of MNPs at the excitation wavelength (532 nm), these structures have been realized with lower exposure time and power, as compared to those required for fabrication with pure SU-8 photoresist. We also demonstrated the the capacity of using the magneto-photonic devices as the free-moving magnetic submicropillars in response to an applied external magnetic field. These results open many promising applications, such as tunable photonic structures based on magneto-optical effect and development of microrobotic tools for transport in biological systems.

## Supplementary Materials

Videos of movement of magnetic nanopillars. The following are available online at http://www.mdpi.com/2079-4991/7/5/105/s1, *Video S.1-Magnetic submicrotructures without external magnetic field*: showing random movement of free-floating magnetic nanopillars immersed in a SU-8 developer solution, without applying external magnetic field. *Video S.2-Magnetic submicrotructures with external magnetic field*: showing directional movement of free-floating magnetic nanopillars, which are immersed in a SU-8 developer solution, when applying an external magnetic field.

## Appendix A

### Appendix A.1. Size and Morphology of the Fe*_3_*O*_4_* Nanoparticles

Figure A1a shows a transmission electron microscopy (TEM) image of ${\mathrm{Fe}}_{3}$$O_{4}$ MNPs in ethanol. The average size of MNPs is determined to be about 11 nm from the TEM image (Figure A1). Due to the inter-particle interactions, the MNPs tend to agglomerate like particle clusters. To minimize the particle agglomeration, the MNPs in ethanol were sonicated for 30 min before incorporating them into a polymer matrix.

### Appendix A.2. Magnetic Characterization of Fe*_3_*O*_4_* Nanoparticles

Magnetic hysteresis loop M(H) was performed on powder ${\mathrm{Fe}}_{3}$$O_{4}$ nanoparticles at room temperature using a vibrating sample magnetometer equipped within the Physical Property Measurement System (PPMS) from Quantum Design. As one can see clearly in Figure A2, the saturation magnetization of the synthesized ${\mathrm{Fe}}_{3}$$O_{4}$ nanoparticles is $M_{S}$ = 63 emu/g, which is smaller compared to bulk ${\mathrm{Fe}}_{3}$$O_{4}$ ($M_{S}$ = 90 emu/g). This can be attributed to the effect of surface spin disorder and the loss of long-range ferromagnetism, both reducing the total magnetization upon particle size reduction to the nanoscale [37]. It is also noted in Figure A2 that there is no hysteresis ($H_{C}∼0$) in the M(H) loop taken at 300 K, indicating the room-temperature superparamagnetic characteristic of the ${\mathrm{Fe}}_{3}$$O_{4}$ nanoparticles. The nature of superparamagnetism is confirmed by the best fit of the M(H) data to the standard Langevin function [38,39]. Since these ${\mathrm{Fe}}_{3}$$O_{4}$ nanoparticles are well dispersed in polymer (SU8 photoresist), the superparamagnetic property of the ${\mathrm{Fe}}_{3}$$O_{4}$ nanoparticles is therefore preserved in the ${\mathrm{Fe}}_{3}$$O_{4}$/polymer nanocomposite, as reported in a previous study [4].

### Appendix A.3. Effects of Viscosity and Mixing Time on Dispersion of Fe*_3_*O*_4_* Nanoparticles in Polymer

In order to incorporate the MNPs with SU-8 photoresist, the magnetic decantation process was repeated to exchange solution from water to ethanol. MNPs in ethanol are treated with ultrasonic wave in a conventional ultrasonic-bath within 30 min before introducing them into a polymer matrix. Different types of epoxy-based negative photoresist, SU-8 2000.5, SU-8 2002, SU-8 2005, SU-8 2010, and SU-8 2025 (MicroChem Corp.) with different viscosities of 2.49 cSt, 7.5 cSt, 45 cSt, 380 cSt, and 4500 cSt, respectively, were used as candidates for hosting MNPs. The mixtures were stirred for 2 h (typical duration) and followed by 30 min of sonication. Finally, nanocomposite solutions of different viscosities were stored in a nonmagnetic environment and observed by optical microscopy as a function of time.

Figure A3 shows the effect of the host environment and the preparation time on the inter-diffusion of ${\mathrm{Fe}}_{3}$$O_{4}$ MNPs in suspension. The ${\mathrm{Fe}}_{3}$$O_{4}$/SU-8 2005 nanocomposite was found to be the best hybrid material, in particular after 20 days of the preparation.
